# Splenic Ly6C^hi^ monocytes contribute to adverse late post-ischemic left ventricular remodeling in heme oxygenase-1 deficient mice

**DOI:** 10.1007/s00395-017-0629-y

**Published:** 2017-05-22

**Authors:** Mateusz Tomczyk, Izabela Kraszewska, Krzysztof Szade, Karolina Bukowska-Strakova, Marco Meloni, Alicja Jozkowicz, Jozef Dulak, Agnieszka Jazwa

**Affiliations:** 1 Department of Medical Biotechnology, Faculty of Biochemistry, Biophysics and Biotechnology, Jagiellonian University, Gronostajowa 7, 30-387 Krakow, Poland; 2 Department of Clinical Immunology and Transplantology, Polish-American Institute of Pediatrics, Jagiellonian University School of Medicine, Krakow, Poland; 3 British Heart Foundation Centre for Cardiovascular Science, University of Edinburgh, Edinburgh, UK; 4 Malopolska Centre of Biotechnology, Jagiellonian University, Krakow, Poland

**Keywords:** Cardiac rupture, Heme oxygenase-1, Macrophages, Monocytes, Myocardial infarction

## Abstract

Heme oxygenase-1 (Hmox1) is a stress-inducible protein crucial in heme catabolism. The end products of its enzymatic activity possess anti-oxidative, anti-apoptotic and anti-inflammatory properties. Cardioprotective effects of Hmox1 were demonstrated in experimental models of myocardial infarction (MI). Nevertheless, its importance in timely resolution of post-ischemic inflammation remains incompletely understood. The aim of this study was to determine the role of Hmox1 in the monocyte/macrophage-mediated cardiac remodeling in a mouse model of MI. Hmox1 knockout (Hmox1^−/−^) and wild-type (WT, Hmox1^+/+^) mice were subjected to a permanent ligation of the left anterior descending coronary artery. Significantly lower incidence of left ventricle (LV) free wall rupture was noted between 3rd and 5th day after MI in Hmox1^−/−^ mice resulting in their better overall survival. Then, starting from 7th until 21st day post-MI a more potent deterioration of LV function was observed in Hmox1^−/−^ than in the surviving Hmox1^+/+^ mice. This was accompanied by higher numbers of Ly6C^hi^ monocytes in peripheral blood, as well as higher expression of monocyte chemoattractant protein-1 and adhesion molecules in the hearts of MI-operated Hmox1^−/−^ mice. Consequently, a greater post-MI monocyte-derived myocardial macrophage infiltration was noted in Hmox1-deficient individuals. Splenectomy decreased the numbers of circulating inflammatory Ly6C^hi^ monocytes in blood, reduced the numbers of proinflammatory cardiac macrophages and significantly improved the post-MI LV function in Hmox1^−/−^ mice. In conclusion, Hmox1 deficiency has divergent consequences in MI. On the one hand, it improves early post-MI survival by decreasing the occurrence of cardiac rupture. Afterwards, however, the hearts of Hmox1-deficient mice undergo adverse late LV remodeling due to overactive and prolonged post-ischemic inflammatory response. We identified spleen as an important source of these cardiovascular complications in Hmox1^−/−^ mice.

**Electronic supplementary material:**

The online version of this article (doi:10.1007/s00395-017-0629-y) contains supplementary material, which is available to authorized users.

## Introduction

Upon myocardial infarction (MI) immune system becomes activated by extensive necrosis of cardiomyocytes and release of damage-associated molecular patterns [16]. Neutrophils are the first immune cells that infiltrate the damaged tissue as early as 24 h after injury. Then, 3–4 days later inflammatory monocytes are recruited into ischemic area of the heart. Apparently, overactive and prolonged immune responses can be responsible for heart failure in patients surviving the ischemic episode [13, 21]. Thus, development of strategies targeting particular subsets of inflammatory cells and providing well-timed resolution of inflammation may be crucial for proper healing and recovery.

Heme oxygenase-1 (Hmox1) catalyzes the conversion of heme to biliverdin, carbon monoxide (CO), and ferrous iron (Fe^2+^). Through the first two compounds Hmox1 mitigates cellular injury. This is accomplished by their antioxidant, anti-apoptotic, and anti-inflammatory effects [7, 34]. We have previously demonstrated the impaired wound healing [9, 20] and recovery after hind limb ischemia [25] in Hmox1 knockout (Hmox1^−/−^) mice when compared to their wild-type (WT, Hmox1^+/+^) littermates. An important role of this protein was also demonstrated in different experimental studies on heart damage. Short-term cardiac stress, induced by ischemia–reperfusion (I/R) injury resulted in impaired left ventricular (LV) recovery and increased infarct size in heterozygous Hmox1-deficient (Hmox1^+/−^) mice [55]. In contrast, transgenic animals with cardiac-specific Hmox1 overexpression were better protected from I/R, with improved contractile recovery and reduced infarct size, inflammatory cell infiltration, oxidative damage, and apoptosis [54]. Recently, *Hmox1* gene transfer was shown to attenuate post-ischemic inflammation in both murine and porcine I/R-injured hearts [22]. In addition, beneficial effects of Hmox1 were demonstrated also under prolonged ischemia in mice with permanent ligation of the coronary artery [47]. Myocyte-restricted Hmox1 transgenic mice exposed to MI exhibited significantly improved survival and LV function, lower interstitial fibrosis and oxidative stress. These beneficial effects were at least partially mediated by CO-dependent inhibition of mitochondrial permeability transition and apoptosis [47].

Importantly, the findings in animal models are in accordance with several clinical investigations, since in humans *HMOX1* gene expression is modulated by a guanidine thymidine dinucleotide ([GT]n) repeat polymorphism in the promoter region (reviewed in: [8]). Shorter repeats with (GT)n <25 are associated with higher inducibility and activity of HMOX1. On the other hand, the longer repeats result in lower HMOX1 expression and activity and were associated with an increased risk of cardiovascular disease (reviewed in: [8]). Thus, heme oxygenase-1 is important for cardioprotection and repair, but its involvement in the suppression of post-ischemic inflammation remains incompletely understood.

Murine blood monocytes are generally divided into two main subpopulations. One is described as non-classical or non-inflammatory (Ly6C^+^CD43^++^; Ly6C^lo^). These cells patrol the intravascular endothelial cell surface and clear dying endothelial cells [37]. Another subset called classical or inflammatory (Ly6C^++^CD43^+^; Ly6C^hi^) spikes during acute MI and accumulates in the evolving myocardial wound [37]. Sometimes, also a third subset is distinguished—intermediate monocytes (Ly6C^++^CD43^++^; also Ly6C^hi^), that are found at a lower frequency and to some extent resemble the classical monocytes, as they expand with cytokine treatment and in inflammation [56]. In contrast to Ly6C^lo^, the Ly6C^hi^ monocytes express on their surface a chemokine receptor CCR2 [43]. Interaction between CCR2 and its ligand CCL2 (monocyte chemoattractant protein-1, Mcp-1), upregulated in the inflamed tissue, results in inflammatory monocyte recruitment [10]. It was recently shown that the post-I/R influx of immune cells, including Ly6C^hi^ monocytes, was further exacerbated in Hmox1-deficient murine hearts [22]. On the other hand, overexpression of *Hmox1* in the heart with gene therapy was able to revert this process [22].

Importantly, under inflammatory conditions monocyte-derived macrophages gradually replenish the population of cardiac-resident macrophages [15]. The latter ones are described as embryonically derived and capable of sustaining themselves by local proliferation [15]. Importantly, their substitution with monocyte-derived macrophages may be responsible for decreased cardiac repair potential [29]. Additionally, mechanical strain rising in the LV wall after MI, evokes macrophage proliferation [41]. It was demonstrated that injection of nanoparticle-loaded siRNA targeting CCR2 to the atherosclerotic apolipoprotein knockout (ApoE^−/−^) mice with MI reduces the recruitment of Ly6C^hi^ monocytes, improves the infarct healing and attenuates the post-MI heart failure [35]. On the other hand, macrophages are important in resolution of inflammation. They produce anti-inflammatory cytokines and chemokines and eliminate tissue debris. In fact, either increased or insufficient macrophage expansion was demonstrated to impair tissue healing after cardiac injury [14]. Evidently, Hmox1 is important for a proper maintenance and function of macrophages [39, 49, 53].

At steady state, monocytes are produced in the bone marrow from hematopoietic precursors, but during inflammation also spleen is involved in generation, depot and deployment of these cells [45]. The organ may host extramedullary hematopoiesis thanks to its ability to adopt hematopoietic stem and progenitor cells (HSPCs) liberated from the bone marrow in response to ischemic insult. Several reports demonstrated an important role of Hmox1 also in differentiation of lineage committed progenitors involved in erythropoiesis, thrombopoiesis, or B cell maturation (reviewed in: [27]). However, involvement of this protein in monocytes generation and release from spleen following MI has not yet been investigated.

Thus, the aims of this study were to determine the role of Hmox1 in post-MI monocytes mobilization and their recruitment to injured cardiac muscle, as well as to check how different populations of cardiac macrophages are affected after MI in the presence and absence of Hmox1.

## Materials and methods

### Experimental animals

For our studies we used 12–14-week-old female Hmox1^+/+^ (WT) and Hmox1^−/−^ specific pathogen-free mice of C57BL/6×FVB background. Age-matched mice were randomly allocated either to sham or MI surgery. All animal procedures were in accordance with *Guide for the Care and Use of Laboratory Animals* (Directive 2010/63/EU of European Parliament) and carried out under a license from the Ethical Committee of the Jagiellonian University. All animals were maintained under controlled environmental conditions (12 h light/dark cycle, at approx. 23 °C), and provided with standard laboratory food and water *ad libitum*.

### Myocardial infarction surgery

Myocardial infarction was induced by permanent ligation of the left anterior descending (LAD) coronary artery as described previously [36]. Mice were anesthetized by intraperitoneal injection of 2,2,2-tribromoethanol (400 mg/kg of body weight). The limb withdrawal response to toe pinch was monitored to ensure the adequacy of anesthesia. Then, mice were placed on temperature-controlled, heated table, intubated and ventilated by connecting the 22G cannula inside the tracheal tube to a mechanical ventilator (MiniVent Type 854; respiration rate: 220 breaths/min, stroke volume 280 µl). The skin and muscles were cut along a breastbone, on the left side of a chest. Incision between 4th and 5th rib was made. Ribs were retracted with surgical threads and heart was exposed. Pericardial sac was opened, respiration rate was reduced to 150 breaths/min and LAD coronary artery was permanently ligated with 7.0 silk suture. Successful LAD occlusion was confirmed by an immediate color change of the myocardium supplied by the vessel from bright red to white. Initial respiration rate was restored and incisions between ribs, muscles and skin were closed with 6.0 silk suture. Mice were disconnected from the lung ventilator to allow normal breathing. In case of sham operated mice the procedure was the same, with placing the suture underneath LAD but without its ligation. Analgesia was applied to all (sham and MI-operated) animals twice daily for 3 consecutive days after surgery by subcutaneous injection of buprenorphine at the dose of 0.08 mg/kg of body weight.

### Splenectomy

During isoflurane anesthesia the abdominal cavity of mice was opened above the left kidney. Then, the spleen vessels were carefully cauterized and spleen was removed. One month later mice were exposed to sham or MI surgeries as described above.

### Detection of cardiac troponin I in plasma

Facial vein puncture was performed intravitally 24 h after each MI or sham surgery to obtain the peripheral blood (PB) anticoagulated with heparin (5 U/ml of blood). After centrifugation (800×*g*, 10 min) plasma was collected and stored frozen at −80 °C until further analysis. Cardiac troponin I (cTnI) was assessed in 100 μl of plasma with ELISA (DRG MedTek) according to vendor’s protocol.

### Detection of hypoxic cells

To assess hypoxia of cardiac muscle Hmox1^+/+^ and Hmox1^−/−^ mice were injected intraperitoneally with 60 mg/kg of body weight of pimonidazole hydrochloride (Hypoxyprobe, HP, Inc., MA, USA) and analyzed as previously described [24, 26]. Thirty minutes after HP injection half of the animals in each group was subjected to LAD ligation. The other half was sham-operated. Then, 90 min after the surgery (and 2 h after HP injection) mice were killed for analysis of hypoxic cells with flow cytometry.

For preparing single cell suspensions from a heart, blood was washed out by injecting saline containing 0.5 U/ml heparin through the LV. Next, the whole heart was excised and both atria carefully cut off. Then, both ventricles were finely minced and incubated in 3 ml mixture of 5 mg/ml of collagenase type II (Gibco) and 1.2 U/ml of dispase (Gibco) in PBS with calcium and magnesium ions (Gibco) for 1 h at 37 °C with gentle agitation. Then, equal volume of Dulbecco’s Modified Eagle’s Medium (DMEM; Gibco) supplemented with 10% Fetal Bovine Serum (FBS; Gibco) was added. Dissociated cells and undigested tissue were centrifuged at 200×*g* for 5 min and the pellet was resuspended in 5 ml of calcium and magnesium free PBS (Gibco). Cell/tissue suspensions were filtered through 100 µm cell strainers, centrifuged and resuspended in PBS containing 2% FBS. Then, the cells were stained for 30 min at 4 °C with 7-AAD (7-amino-actinomycin D) for the exclusion of nonviable cells. Next, the cells were washed with PBS, fixed and permeabilized for 20 min at room temperature (RT) with Cytofix/Cytoperm (BD Biosciences) according to vendor’s protocol. After washing, the cells were incubated with FITC-conjugated anti-HP antibody (Clone: 4.3.11.3, Hypoxyprobe, Inc., Massachusetts, USA) detecting protein adducts of pimonidazole in hypoxic tissue. The stained cells were analyzed using LSRFortessa cytometer (BD Biosciences) and Facs Diva (BD Biosciences) Software.

### Transthoracic echocardiography

Mice were subjected to inhalation anesthesia with 2% isoflurane (Aerrane, Baxter) in air and immobilized on a heating platform ventral side up to maintain the body temperature at 37 ± 0.5 °C. Heart rate and respiration were continuously monitored by ECG electrodes. Mice chests were shaved and pre-warmed ultrasound gel was applied to the area of interest. Transthoracic echocardiography (TTE) was performed on day 7, 14 and 21 after MI using a Vevo 2100 system (Visual Sonics, Canada) with a 30-MHz transducer. The heart was first imaged in B-mode in the parasternal long axis (PSLA) view to examine the left ventricle (LV). Moreover, parasternal short axis (SAX) view was obtained at the level of papillary muscles. In case of each animal two-dimensional (2D) images crossing the anterior and posterior walls in PSLA and SAX were recorded. The following parameters were investigated: LV ejection fraction (LV EF, %), LV fractional shortening (LV FS, %), LV chamber volume (LV V, μl) and LV internal diameter (LV ID, mm) during both systole (s) and diastole (d).

### Flow cytometry

At indicated time-points mice were anesthetized with 2% isoflurane (Aerrane, Baxter) in air and few drops of blood were collected by facial vein puncture to Microvette 100 EDTA tubes (Sarstedt) for white blood cell (WBC) count analysis using ABC Vet Hematology Analyzer (Horiba). Next, mice were euthanized by intraperitoneal injection of a mixture of ketamine (200 mg/kg of body weight) and xylazine (40 mg/kg of body weight). Peripheral blood was collected with a heparinized syringe from vena cava. Then, the animal was perfused with 5 ml saline containing 0.5 U/ml heparin through the LV. Heart, spleen, as well as femora and tibiae from both hind limbs were collected. Cells were isolated as described below.

#### Peripheral blood

Heparin blood samples (approx. 500–700 µl) were centrifuged (4 °C, 800×*g*, 10 min) and plasma was collected and stored frozen at −80 °C until further analysis. Then, all morphotic elements were suspended in 15 ml of hypotonic 1× RBC lysis buffer (formulation of working solution: 150 mM NH_4_Cl, 10 mM NaHCO_3_, 1 mM disodium EDTA, pH 7.4) for red blood cell (RBC) lysis and then centrifuged. If necessary, the lysis was repeated. After complete RBC lysis and removal of supernatant, cells were washed with PBS.

#### Spleen

The organ was cut in large pieces and pressed against a 100 µm cell strainer. Cells were washed with PBS and centrifuged. Then, the supernatant was discarded. Red blood cells were removed as described above. After complete RBC lysis and removal of supernatant, cells were washed with PBS.

#### Heart

Single cell suspensions from both ventricles of the heart were prepared as described in “Detection of hypoxic cells”. Red blood cells were removed as described above. After complete RBC lysis and removal of supernatant, cells were washed with PBS.

#### Bone marrow

Epiphyses of femora and tibiae were cut and bone marrow was flushed from bones with PBS using a syringe with 30 gauge needle. Next, cells were centrifuged and red blood cells were removed as described above. After complete RBC lysis and removal of supernatant, cells were washed with PBS.

Before staining cells were incubated for 10 min on ice in PBS containing 2% FBS and anti-mouse CD16/CD32 antibody (clone 93, eBioscience) in concentration recommended by the vendor. Finally, cells were mixed with adequate panel of antibodies (Suppl. Table 1 and Suppl. Table 2) and stained for 25 min at 4 °C. For monocyte and macrophage staining, cell suspensions were incubated with antibodies directed against CD45, Ly6G, Ly6C, NK1.1, CD11b, CD11c, CD43, MHC II. For HSPCs, cell suspensions were incubated with antibodies directed against lineage markers (Ter119, CD3, B220, CD11b, Gr-1), Sca-1, CD117 (c-kit), CD34, CD48, CD150 (SLAM). In all staining procedures live cells were analyzed and dead cells were excluded by DAPI staining. Gating strategies for blood and spleen monocytes (Suppl. Figure 1), cardiac monocytes and macrophages (Suppl. Figure 2) and HSPCs in bone marrow and spleen (Suppl. Figure 3) are enclosed to Supplemental Material. The stained cells were analyzed using LSRFortessa cytometer (BD Biosciences) and Facs Diva (BD Biosciences) Software.

### Western blotting

Pieces of tissue from infarct area and corresponding heart tissue from sham-operated mice (*n* = 2 mice/group) were collected on day 4, snap-frozen in dry ice and stored at −80 °C. Total protein was isolated by lysis of tissue in 300 µl of lysis buffer (1% Triton X-100 in PBS with protease inhibitors). Homogenization was performed using Tissue Lyzer (Qiagen). Protein concentration was determined using bicinchonic acid assay (BCA). For collagen type I detection, samples containing 40 µg of protein were prepared in non-reducing conditions and resolved in 7.5% SDS-polyacrylamide gel electrophoresis (SDS-PAGE) and transferred to nitrocellulose membrane (wet transfer, 80 V, 2 h). For α-tubulin detection, samples were prepared in reducing conditions and subjected to the same procedure as described above. Membranes were blocked with 5% bovine serum albumin (BSA) in PBS containing 0.05% Tween-20 (collagen type I) or 5% milk in PBS containing 0.05% Tween-20 (α-tubulin) for 2 h at room temperature. Then, primary antibodies were added in 1:500 dilution (collagen type I, Abcam, ab21286) or 1:1000 dilution (α-tubulin, Calbiochem, DM1A) in blocking buffer and membranes were incubated overnight at 4 °C. On the following day, membranes were washed 5 times for 5 min with PBS containing 0.1% Tween-20. In the next step, secondary antibodies conjugated with horseradish peroxidase were added in 1:10,000 dilution in blocking buffer for 45 min at room temperature. After washing steps, chemiluminescent substrate for HRP activity (Immobilon Western Chemiluminescent HRP substrate, Merck Millipore) was added for 5 min and membranes were developed on photographic membranes.

### Samples collection and histological examination

Histological analyses were performed in frozen sections of the hearts collected from mice 21 days after MI (*n* = 3 mice/group). The chest of anesthetized mice was opened and the heart arrested in diastole by intraventricular (LV) injection of 30 mM KCl in saline containing 0.5 U/ml of heparin. The right atrium was then cut and the heart was perfused with 5 ml of saline supplemented with 0.5 U/ml of heparin at a pressure similar to the mean arterial pressure via PE-50 catheter connected to a perfusion apparatus. After excision, heart was sliced into two transversal sections at the site of LAD ligation. The part below LAD ligation was embedded in OCT compound (Tissue-Tek) and snap-frozen in dry ice.

#### Assessment of cardiomyocyte hypertrophy

Sections were blocked with 1% BSA in PBS (1 h, RT) and then incubated with rhodamine-labeled wheat germ agglutinin (WGA; Vector Laboratories; dilution: 1:100, 30 min, RT). Sections were mounted with DAPI (4′,6-diamidino-2-phenylindole) containing medium to visualize nuclei. Analysis was performed at a 200× magnification using the ImageJ software by an observer blinded to the experimental protocol. For each sample, cardiomyocyte cross sectional area (CSA) was measured in 100 cardiomyocytes in which the nucleus was centrally located within the cell, in both the border peri-infarct zone and in the remote zone. The average regional cross-sectional area in μm^2^ were calculated.

#### Assessment of CD11b^+^ myeloid cells

Sections were blocked with PBS solution containing 10% goat serum, 1% BSA and 0.1% Triton (1 h 30 min, RT). After blocking tissue sections were incubated with primary rat anti-mouse CD11b antibody (BioRad, clone: 5C6; dilution: 1:200, overnight 4 °C). After washing in PBS the secondary anti-rat antibody (Alexa Fluor 488) was applied (dilution: 1:400, 1 h 30 min, RT). Sections were mounted with DAPI-containing medium to visualize nuclei. The cells were visualized in the peri-infarct zone at a 200× magnification.

### Total RNA isolation, reverse transcription and quantitative PCR

Pieces of tissue from infarct area of the hearts and corresponding tissue samples from sham-operated and untreated mice were collected, snap-frozen in dry ice and stored at −80 °C. Total RNA isolation by lysis in 1 ml of Qiazol (Qiagen) per tissue sample was performed using Tissue Lyzer (Qiagen) followed by chloroform extraction and isopropanol precipitation. Concentration and quality of RNA was determined by 260 and 280 nm absorbance measurements using NanoDrop Spectrophotometer (Thermo Fischer Scientific). For cDNA synthesis 1 μg of RNA was used. Using NCode miRNA First-Strand cDNA Synthesis Kit (Invitrogen) cDNA was synthesized in total volume of 15 μl, according to vendor’s protocol. Obtained template for quantitative PCR (qPCR) was ten times diluted in ultrapure water. Quantitative PCR (qPCR) was performed using StepOne Plus Real-Time PCR (Applied Biosystems) with addition of SYBR Green PCR Master Mix (SYBR^®^ Green JumpStart™ *Taq*, Sigma), 2 μl of diluted template and specific primers (Suppl. Table 3). Elongation factor 2 (*Ef2*) served as a housekeeping gene in mRNA analyses. In test samples, quantification of gene expression was calculated based on the comparative CT (threshold cycle value) method (ΔCT = CT gene of interest − CT housekeeping gene). The melting curve analysis was performed immediately after each qPCR for the presence of primer dimers or non-specific products.

### Statistical analyses

Statistical analyses were carried out using GraphPad Prism software version 5 (GraphPad Software, Inc.). Results are expressed as mean ± SEM unless otherwise stated. First, values were tested for Gaussian distribution (Kolmogorov–Smirnov test). For two-group comparisons, an unpaired *t* test was applied to normally distributed variables, a Mann–Whitney test to non-normally distributed variables. For comparing more than two groups, a one-way ANOVA followed by Bonferroni’s post hoc test for multiple comparisons, was applied. Survival curves were analyzed by Log-rank test. *p* < 0.05 was considered statistically significant.

## Results

### Hmox1 deficiency differently influences early and late post-MI cardiac remodeling

**Fig. 1:**
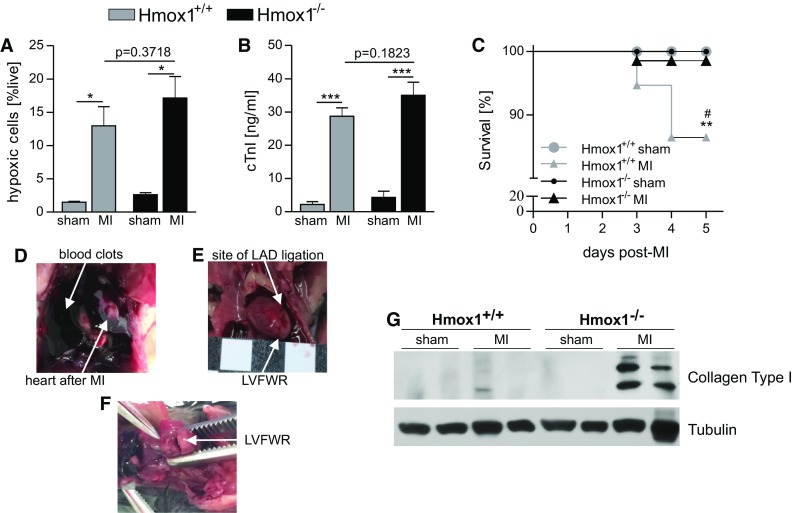
Lower early post-MI survival in WT than in Hmox1-deficient mice. **a** Flow cytometric detection of cells labeled with pimonidazole hydrochloride in the heart 90 min after LAD ligation (*n* = 4 mice/group). **b** ELISA for cTnI in plasma one day after LAD ligation (sham: *n* = 26 mice/group, MI: *n* = 36 mice/group). **c** Kaplan–Meier survival curves of Hmox1^+/+^ and Hmox1^−/−^ mice subjected to sham and MI surgeries (sham: *n* = 18 mice/group, MI: *n* = 32–37 mice/group). *p < 0.05, **p < 0.01, ***p < 0.0001; *vs. appropriate sham control, ^#^vs. other MI. **d**–**f** Photographs of an autopsied mouse that died due to cardiac rupture. **d** The chest cavity was filled with a large amount of clotted blood. **e**, **f** Photographs of the heart with a left ventricular perforation in the area of apex. *MI* myocardial infarction, *LAD* left anterior descending coronary artery, *LVFWR* left ventricular free wall rupture. **g** Western blot analysis of collagen type I in heart on day 4 after surgery

Hmox1-deprived mice and their WT littermates were subjected to either LAD ligation or sham surgery. We used pimonidazole hydrochloride to evaluate hypoxia in cardiac tissue 90 min after surgery. This compound is reductively activated and forms adducts with thiol groups of macromolecules at low oxygen levels [46]. Detection with flow cytometry revealed that in both genotypes MI significantly increased the number of cells positive for hypoxic marker (pimonidazole-protein adducts) when compared to sham-operation and no difference was observed between Hmox1^−/−^ and WT mice subjected to LAD ligation (Fig. 1a). In addition, highly increased, when compared to sham controls, plasma cardiac troponin I (cTnI) levels were detected by ELISA on first day after MI (Fig. 1b). We did not observe any cTnI circulating in the blood of intact animals (not shown). There was no significant difference between post-MI cTnI levels in Hmox1^−/−^ and Hmox1^+/+^ mice (Fig. 1b).

Survival of mice after operation was monitored until the end of each experiment (maximum of 21 days). We noticed that all demises occurred only between 3rd and 5th day after LAD ligation (Fig. 1c). Survival rate of sham-operated mice, independently of the genotype, was 100%. A significantly higher mortality post-MI was observed in WT mice compared to Hmox1^−/−^ (Fig. 1c). Cause of death was identified as an internal bleeding associated with left ventricular free wall rupture (LVFWR) below the site of coronary artery ligation. This was confirmed during necropsy (Fig. 1d–f). In relation to this we checked the collagen content, as it was previously demonstrated to be inversely correlated with the incidence of rupture [5, 18, 19, 50]. Western blot analysis on day 4 post-MI revealed higher production of collagen type I in the infarcted area of the heart of Hmox1-deficient mice than of their WT littermates (Fig. 1g).

**Fig. 2:**
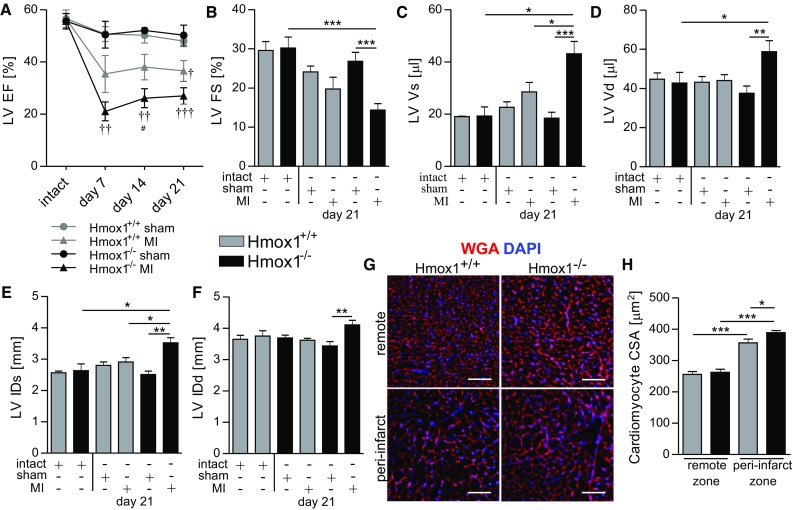
More profound LV dysfunction in Hmox1^−/−^ than in Hmox1^+/+^ mice after MI. At indicated time-points after MI or sham surgery **a** LV EF, **b** LV FS, **c** LV Vs, **d** LV Vd, **e** LV IDs, and **f** LV IDd were determined with TTE; *n* = 7–10 mice/group. **g**, **h** Evaluation of cardiomyocyte hypertrophy. **g** Representative fluorescent images of WGA (*red*) and DAPI (*blue*) in the peri-infarct (border zone) and remote myocardium (remote zone) of MI-operated Hmox1^+/+^ and Hmox1^−/−^ mice. **h** Graph summarizing quantitative analysis of cardiomyocyte cross-sectional area (CSA) in the border and remote myocardium.*p < 0.05, **p < 0.01, ***p < 0.0001, ^†^vs. appropriate sham control, ^#^vs. other MI. *Scale bar* 50 μm

TTE revealed reduction of LV EF 7, 14 and 21 days after MI surgery in mice of both genotypes (compared to sham-operated individuals), but more severe deterioration was observed in Hmox1^−/−^ animals (Fig. 2a). In addition, the Hmox1-deprived mice with MI had significantly reduced LV FS (Fig. 2b), increased LV Vs and LV Vd (Fig. 2c, d, respectively), as well as increased LV ID both in systole (Fig. 2e) and diastole (Fig. 2f), when compared to intact and sham-operated Hmox1^−/−^ mice.

Cardiomyocyte cell size assessment using WGA staining showed that in both genotypes on 21st day post-MI cardiomyocyte size was greater in the peri-infarct than in the remote zone of the LV (Fig. 2g, h). This late remodeling response in the peri-infarct region was more pronounced in Hmox1^−/−^ mice than in their WT littermates (Fig. 2h).

### Increased steady state numbers and post-MI monocyte mobilization in the absence of Hmox1

**Fig. 3:**
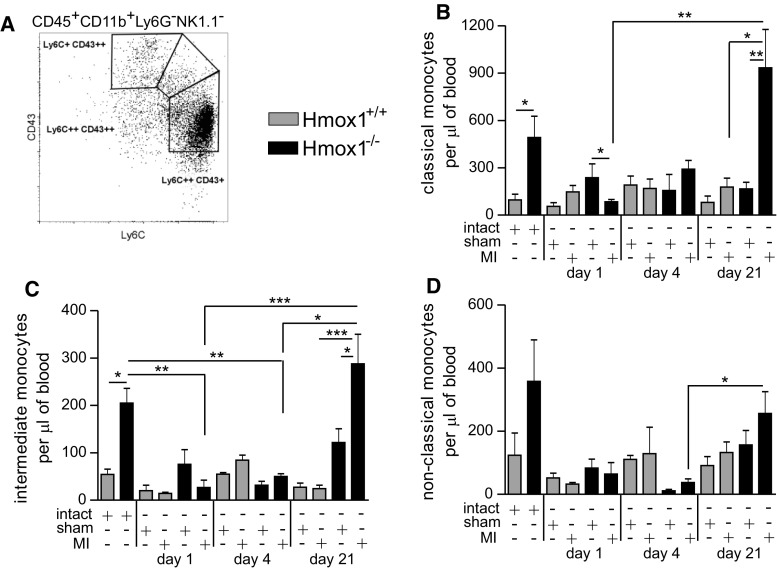
Increased steady state and post-MI numbers of monocytes in the absence of Hmox1. **a** Schematic representation of different populations of blood monocytes based on the expression of CD43 and Ly6C markers. Flow cytometric analysis of **b** classical monocytes (CD45^+^ CD11b^+^ Ly6G^−^ NK1.1^−^ Ly6C^++^ CD43^+^), **c** intermediate monocytes (CD45^+^ CD11b^+^ Ly6G^−^ NK1.1^−^ Ly6C^++^ CD43^++^) and **d** non-classical monocytes (CD45^+^ CD11b^+^ Ly6G^−^ NK1.1^−^ Ly6C^+^ CD43^++^) in the peripheral blood of mice after LAD ligation or sham surgery (*n* = 4–9 mice per group). Data represented as a number of cells per 1 µl of peripheral blood. *p < 0.05, **p < 0.01, ***p < 0.0001

Using flow cytometry, we checked the content of the three major monocyte subsets in peripheral blood following MI. Monocytes were gated as presented in Fig. 3a and complete gating strategy is depicted in Suppl. Figure 1. Steady state numbers of classical (Fig. 3b) and intermediate (Fig. 3c) monocytes in Hmox1^−/−^ mice were approximately twice as high as those observed in their WT littermates. In Hmox1^+/+^ mice, the numbers of classical monocytes did not change much throughout the course of experiment (Fig. 3b). In contrast, the numbers of classical monocytes in the blood of Hmox1^−/−^ mice were initially lower (on first day after MI) and then increased over time reaching over a 3-fold increase when compared to appropriate sham controls on day 21 post-MI (Fig. 3b). Similar pattern was observed for intermediate monocytes (Fig. 3c). The numbers of non-classical blood monocytes were comparable between different experimental groups and only in Hmox1-deficient mice they were transiently decreased on 4th day post-MI (Fig. 3d).

### MI-induced Hmox1 expression is associated with downregulation of genes involved in inflammatory cell infiltration

**Fig. 4:**
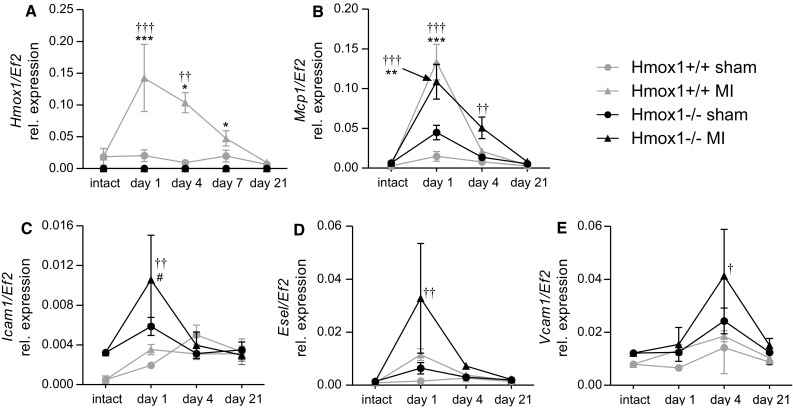
Expression of genes involved in inflammatory cell infiltration inversely correlates with Hmox1 expression. The qPCR for **a** *Hmox1*, **b** *Mcp1*, **c** *Icam1*, **d** *Esel*, **e** *Vcam1* in the infarcted area (or corresponding area in sham-operated and intact controls) of the heart at indicated time points after surgery (*n* = 3–6 mice/group). *p < 0.05, **p < 0.01, ***p < 0.0001; *vs. appropriate sham control, ^†^vs. appropriate intact control, ^#^vs. other MI

As early as 1 day after induction of MI a robust upregulation of *Hmox1* gene expression was observed in the infarct region of the heart of WT mice. *Hmox1* expression then decreased with time, reaching basal levels around 21st day (Fig. 4a). There were no significant changes in the expression of other genes related to iron metabolism, such as ferritin heavy chain (*Fth1*) and heme oxygenase-2 (*Hmox2*) following LAD ligation (data not shown).

One day after MI there was a robust and comparable in both genotypes upregulation of *Mcp1* in the infarcted region of the heart (Fig. 4b). Its elevated levels declined with time and reached the baseline around 21st day post-MI. Importantly, in case of Hmox1-deficient mice, this decrease was delayed and on day 4, in contrast to MI-operated WT mice, there was still a pronounced level of *Mcp*-*1* transcript detected in Hmox1^−/−^ mice with MI (Fig. 4b; day 1 vs. day 4 post-MI in Hmox1^−/−^: *p* = 0.11; day 1 vs. day 4 post-MI in Hmox1^+/+^: *p* < 0.01).

Several leukocyte receptors and vascular adhesion ligands enable leukocyte binding to and passing through the endothelial layer of blood vessels inside the tissue [3]. We checked the expression of three adhesion molecules enabling tethering, rolling and stable adherence of the leukocyte with the subsequent extravasation between endothelial cells. On day 1, post-MI, the mRNA levels of intercellular adhesion molecule 1 (*Icam1*) were significantly higher in the cardiac infarcted area of Hmox1^−/−^ mice than their WT littermates (Fig. 4c). Similar trend was observed for E-selectin (*Esel*) (Fig. 4d) and vascular cell adhesion molecule 1 (*Vcam1*) (Fig. 4e), the later one showing different kinetics with a peak expression on day 4.

### Lack of Hmox1 is associated with increased numbers of monocyte-derived cardiac macrophages following MI

**Fig. 5:**
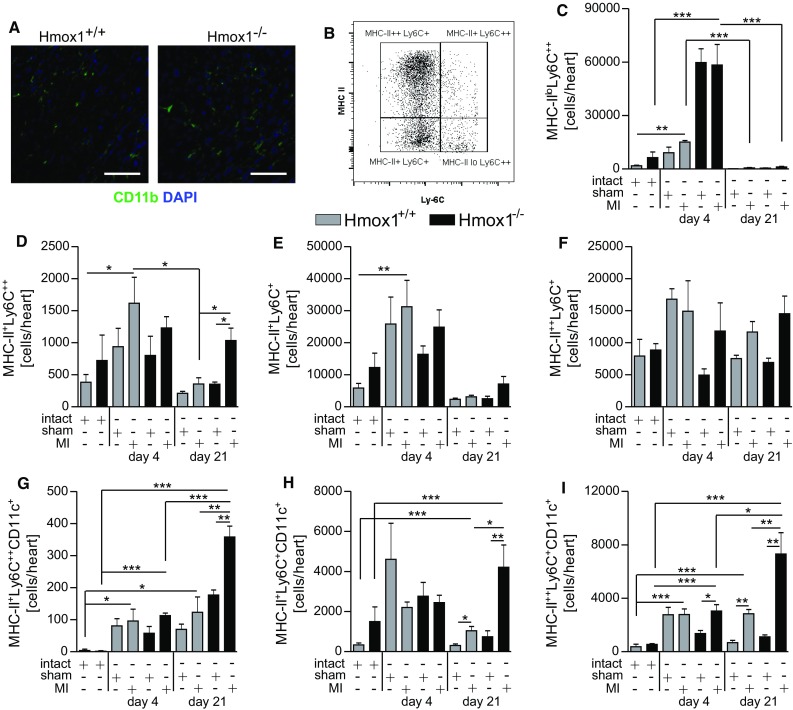
Lack of Hmox1 affects composition of macrophages in ischemic cardiac muscle. **a** Representative fluorescent images of CD11b (*green*) and 4′,6-diamidino-2-phenylindole (DAPI; *blue*) in the peri-infarct region of MI-operated mice on day 21 after surgery. *Scale bar* 50 μm. **b** Schematic representation of different populations of cardiac macrophages and monocytes based on the expression of MHC II and Ly6C markers. Flow cytometric analysis of CD45^+^ Ly6G^−^ CD11b^+^ **c** monocytes (MHC-II^lo^ Ly6C^++^) and different subsets of macrophages: **d** MHC-II^+^ Ly6C^++^, **e** MHC-II^+^ Ly6C^+^, **f** MHC-II^++^ Ly6C^+^, **g** MHC-II^+^ Ly6C^++^ CD11c^+^, **h** MHC-II^+^ Ly6C^+^ CD11c^+^, **i** MHC-II^++^ Ly6C^+^ CD11c^+^ in the myocardium of mice after LAD ligation or sham surgery (*n* = 3–6 mice/group) in indicated time points. Data represented as a number of cells detected in heart. *p < 0.05, **p < 0.01, ***p < 0.0001

Immunofluorescent detection of CD11b^+^ cells revealed that on day 21 post-MI, a vast majority of them accumulated in the peri-infarct area of the LV (Fig. 5a). Monocytes and different populations of macrophages were determined in single-cell suspensions prepared from the hearts using flow cytometry. The CD45^+^ Ly6G^−^ NK1.1^−^ CD11b^+^ cells were gated (complete gating strategy is shown in Suppl. Figure 2). Then, additional markers, MHC-II and Ly6C, allowed us to distinguish populations of monocytes (MHC-II^lo^ Ly6C^+^) and macrophages (MHC-II^+^ Ly6C^+^, MHC-II^++^ Ly6C^+^ and MHC-II^+^ Ly6C^++^) in the heart (Fig. 5b). Independently of the genotype, there was an increase in the number of monocytes detected in cardiac muscle 4 days following ischemic insult (Fig. 5c). Then, on 21st day their numbers decreased (Fig. 5c). Regarding macrophages, the numbers of all investigated subpopulations were comparable in the hearts of intact mice of both genotypes (Fig. 5d–f). Interestingly, in the hearts of Hmox1^−/−^ mice on day 21 post-MI, we detected increased numbers of MHC-II^+^ Ly6C^++^ macrophage subset (Fig. 5d). In the hearts of Hmox1^+/+^ mice, both on day 4 and 21 after surgery, MHC-II^+^ Ly6C^++^ macrophages were found with comparable frequency in sham and MI-operated individuals (Fig. 5d). The MHC-II^+^ Ly6C^+^subset (Fig. 5e) increased on day 4 post-MI only in WT mice, whereas MHC-II^++^ Ly6C^+^ subset (Fig. 5f) did not differ significantly on day 4 and 21 post-MI neither in Hmox1^+/+^ nor Hmox1^−/−^ mice.

Additionally, we found that among MHC-II^+^ Ly6C^++^ (Fig. 5g), MHC-II^+^ Ly6C^+^ (Fig. 5h) and MHC-II^++^ Ly-6C^+^ (Fig. 5i) cardiac macrophage populations, CD11c^+^ subsets expanded. Significant differences were observed between sham- and MI-operated Hmox1^−/−^ mice (Fig. 5i, day 4; g–i, day 21). Less pronounced increases were found between sham- and MI-operated Hmox1^+/+^ mice (Fig. 5h, i, day 21).

### More active hematopoiesis in the bone marrow of Hmox1^−/−^ mice at steady state and after injury

**Fig. 6:**
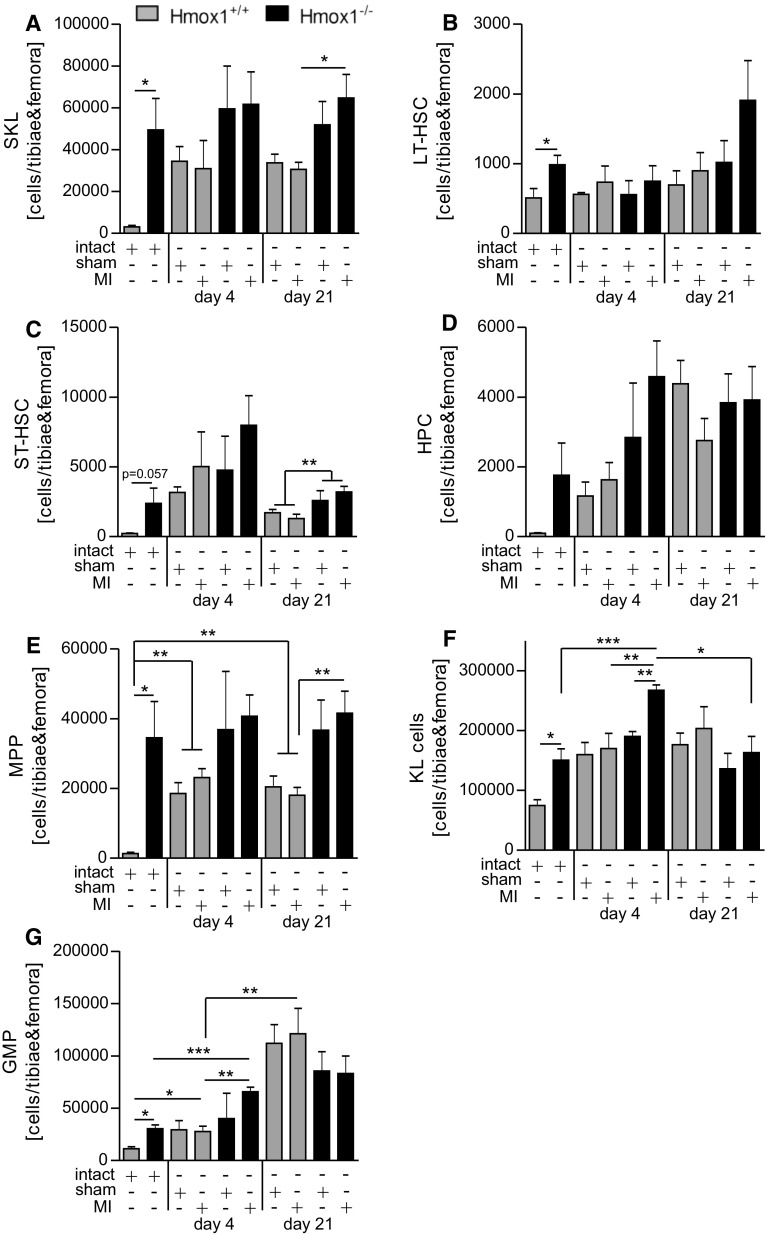
Lack of Hmox1 is associated with higher steady state and post-MI numbers of selected hematopoietic stem and progenitor cell populations in bone marrow. Flow cytometric analysis of **a** SKL cells (CD45^+^ Sca-1^+^ c-kit^+^ Lin^−^), **b** long-term HSC (LT-HSC; CD45^+^ Sca-1^+^ c-kit^+^ Lin^−^ CD34^−^ CD48^−^ CD150^+^), **c** short-term HSC (ST-HSC; CD45^+^ Sca-1^+^ c-kit^+^ Lin^−^ CD34^+^ CD48^−^ CD150^+^), **d** hematopoietic progenitors (HPC; CD45^+^ Sca-1^+^ c-kit^+^ Lin^−^ CD34^+^ CD48^−^ CD150^−^), **e** multipotent hematopoietic progenitors (MPP; CD45^+^ Sca-1^+^ c-kit^+^ Lin^−^ CD34^+^ CD48^+^ CD150^−^), **f** progenitor cells lacking Sca-1 (KL; CD45^+^ Sca-1^−^ c-kit^+^ Lin^−^), **g** granulocyte-monocyte progenitor cells (GMP; CD45^+^ Sca-1^−^ c-kit^+^ Lin^−^ CD34^+^ CD48^++^ CD150^−^) in the bone marrow of mice after LAD ligation or sham surgery (*n* = 4–10 mice/group). Data represented as a number of cells in bone marrow isolated from tibiae and femora. *p < 0.05, **p < 0.01, ***p < 0.0001

Next, we investigated the first putative source of monocytes—the bone marrow. Several cell populations at different levels of the hematopoietic hierarchy were analyzed (Suppl. Figure 3 shows detailed gating strategy). In general, all inspected HSPC populations were more numerous in the bone marrow of Hmox1^−/−^ mice (Fig. 6a–g). By definition, all functional hematopoietic stem cells (HSCs) are described as the population of bone marrow cells that does not express the cell-surface markers normally present on lineage (Lin) committed hematopoietic cells, but does express high levels of stem-cell antigen 1 (Sca-1) and c-kit [51]. In the bone marrow, the number of SKL cells (Sca-1^+^ c-kit^+^ Lin^−^; Fig. 6a) increased on day 4 and remained elevated up to day 21 independently of the surgical procedure (sham or MI) in Hmox1^+/+^ mice, whereas in Hmox1^−/−^ individuals similar numbers of these cells were found throughout the course of the experiment. Nevertheless, at the end of experiment (day 21) the number of SKL cells was higher in Hmox1^−/−^ mice compared to their WT littermates subjected to MI. Because only some phenotypic SKL have long-term repopulating activity, they can be further subdivided into long-term (LT)-HSC and short-term (ST)-HSC, which have only limited self-renewal activity [51]. Among SKL cells, the number of LT-HSC (SKL CD34^−^ CD48^−^ CD150^+^; Fig. 6b) was higher in intact Hmox1^−/−^ than in intact WT mice, however, their numbers were not significantly changed after MI in both genotypes. Higher ST-HSC (SKL CD34^+^ CD48^−^ CD150^+^; Fig. 6c) numbers were detected in Hmox1^−/−^ than in WT mice (intact and on day 21 independently of the surgical procedure). Higher numbers of hematopoietic progenitor cells (SKL CD34^+^ CD48^−^ CD150^−^; HPC; Fig. 6d) and multipotent progenitors (SKL CD34^+^ CD48^+^ CD150^−^; MPP; Fig. 6e) were detected in WT mice on day 4 and 21 independently of the surgical procedure (sham or MI) when compared to intact WT individuals. On day 21 post-MI, MPPs were more frequently detected in the bone marrow of Hmox1^−/−^ than WT mice (Fig. 6e, day 21). The numbers of more differentiated hematopoietic progenitors (Sca-1^−^ c-kit^+^ Lin^−^; KL, Fig. 6f) were transiently increased in Hmox1^−/−^ mice on day 4 and a decline was observed on day 21 post-MI (Fig. 6f). Importantly, the numbers of granulocyte-monocyte progenitors (CD34^+^ CD48^++^ CD150^−^; GMP; Fig. 6g) increased during the course of the experiment in both genotypes. However, significant difference between Hmox1^+/+^ and Hmox1^−/−^ mice subjected to MI was observed only on day 4 (Fig. 6g).

### Generation and release of Ly6C^hi^ monocytes from the spleen of Hmox1^−/−^ mice in response to cardiac injury

**Fig. 7:**
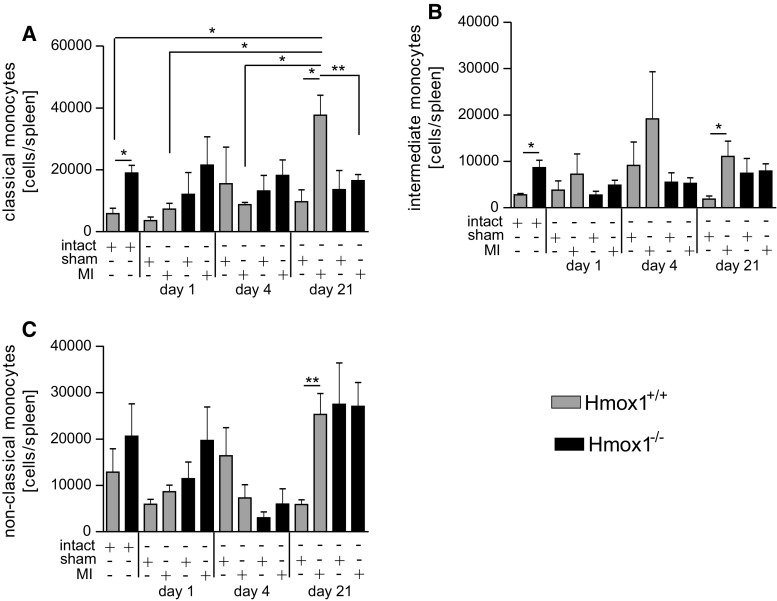
Increased steady state and decreased late post-MI monocyte numbers in spleens of Hmox1^−/−^ mice. Flow cytometric analysis of **a** classical (CD45^+^ CD11b^+^ Ly6G^−^ NK1.1^−^ Ly6C^++^ CD43^+^), **b** intermediate (CD45^+^ CD11b^+^ Ly6G^−^ NK1.1^−^ Ly6C^++^ CD43^++^) and **c** non-classical (CD45^+^ CD11b^+^ Ly6G^−^ NK1.1^−^ Ly6C^+^ CD43^++^) monocytes in the spleens of mice after LAD ligation or sham surgery (*n* = 4–10 mice/group). Data represented as a number of cells per spleen. *p < 0.05, **p < 0.01, ***p < 0.0001

Previously, it was shown that approximately 50% of all monocytes recruited to the heart derive from a splenic reservoir [45]. A detailed analysis of different populations of splenic monocytes revealed significantly higher numbers of classical and intermediate monocyte subsets in intact Hmox1^−/−^ mice than in their intact WT littermates (Fig. 7a, b). The numbers of classical monocytes in the spleens of Hmox1^+/+^ mice were the highest in the end of experiment (Fig. 7a, day 21). Additionally, a significantly lower number of these cells was noted in the spleens of Hmox1^−/−^ mice with MI when compared to MI-operated WT individuals (Fig. 7a, day 21). Non-classical monocytes in the spleen did not differ significantly after LAD ligation in Hmox1^−/−^ mice at any of the time-points analyzed, whereas in WT we observed a significant difference between sham- and MI-operated individuals on day 21 (Fig. 7c).

**Fig. 8:**
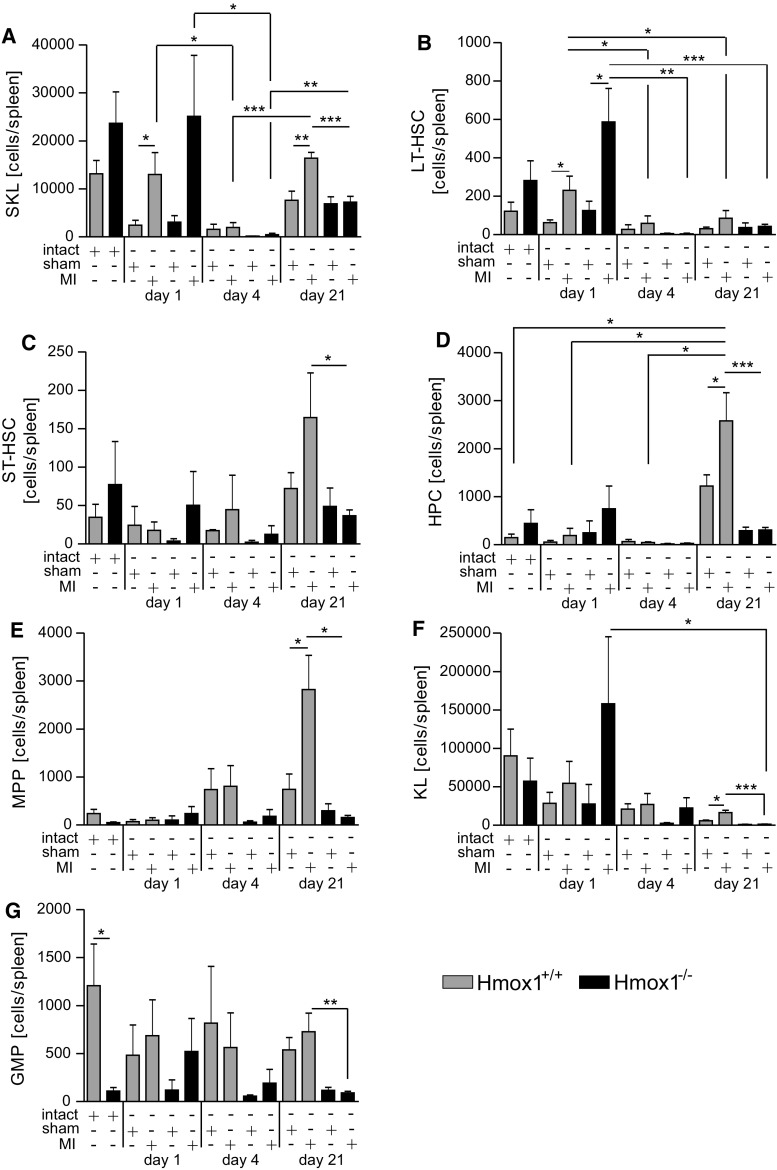
Lower numbers of selected hematopoietic stem and progenitor cell populations in spleens of Hmox1^−/−^ mice 21 days after MI. Flow cytometric analysis of **a** SKL cells (CD45^+^ Sca-1^+^ c-kit^+^ Lin^−^), **b** long-term HSC (LT-HSC; CD45^+^ Sca-1^+^ c-kit^+^ Lin^−^ CD34^−^ CD48^−^ CD150^+^), **c** short-term HSC (ST-HSC; CD45^+^ Sca-1^+^ c-kit^+^ Lin^−^ CD34^+^ CD48^−^ CD150^+^), **d** hematopoietic progenitors (HPC; CD45^+^ Sca-1^+^ c-kit^+^ Lin^−^ CD34^+^ CD48^−^ CD150^−^), **e** multipotent hematopoietic progenitors (MPP; CD45^+^ Sca-1^+^ c-kit^+^ Lin^−^ CD34^+^ CD48^+^ CD150^−^), **f** progenitor cells lacking Sca-1 (KL; CD45^+^ Sca-1^−^ c-kit^+^ Lin^−^) and **g** granulocyte-monocyte progenitor cells (GMP; CD45^+^ Sca-1^−^ c-kit^+^ Lin^−^ CD34^+^ CD48^++^ CD150^−^) in the spleens of mice after LAD ligation or sham surgery (5–10 mice/group). Data represented as a number of cells per spleen. *p < 0.05, **p < 0.01, ***p < 0.0001

Spleen is able to support hematopoietic function of the bone marrow [11, 40]. Thus, we analyzed different populations of HSPCs also in the spleen. After MI, numbers of SKL cells in spleen changed over time. In spleens of mice of both genotypes subjected to LAD ligation, on first day there was an increase of SKLs, and then, on day 4, this population significantly declined in number. On day 21 SKLs re-appeared in both WT and Hmox1^−/−^ mice, but the significant difference between sham- and MI-operated individuals was noticed only for Hmox1^+/+^ mice (Fig. 8a). Among SKLs, similar pattern (apart from day 21) was observed for LT-HSCs (Fig. 8b). However, especially at steady state, these cells were very rare in the spleen (Fig. 8b). On day 21 ST-HSCs (Fig. 8c), HPCs (Fig. 8d) and MPPs (Fig. 8e), expanded after MI in WT mice, whereas their numbers were comparable between sham- and MI-operated Hmox1^−/−^ individuals. Similarly, more differentiated progenitors (KL cells, Sca-1^−^ c-kit^+^ Lin^−^, Fig. 8f) and GMPs (Fig. 8g) were in general less numerous in the spleens of Hmox1^−/−^ mice on day 21 post-surgery (both sham and MI) when compared to corresponding WT mice. Similar genotype-dependent difference was observed also in steady-state numbers of GMPs (Fig. 8g).

### Splenectomy differently influences the post-MI monocyte/macrophage numbers and LV function in Hmox1^+/+^ and Hmox1^−/−^ mice

**Fig. 9:**
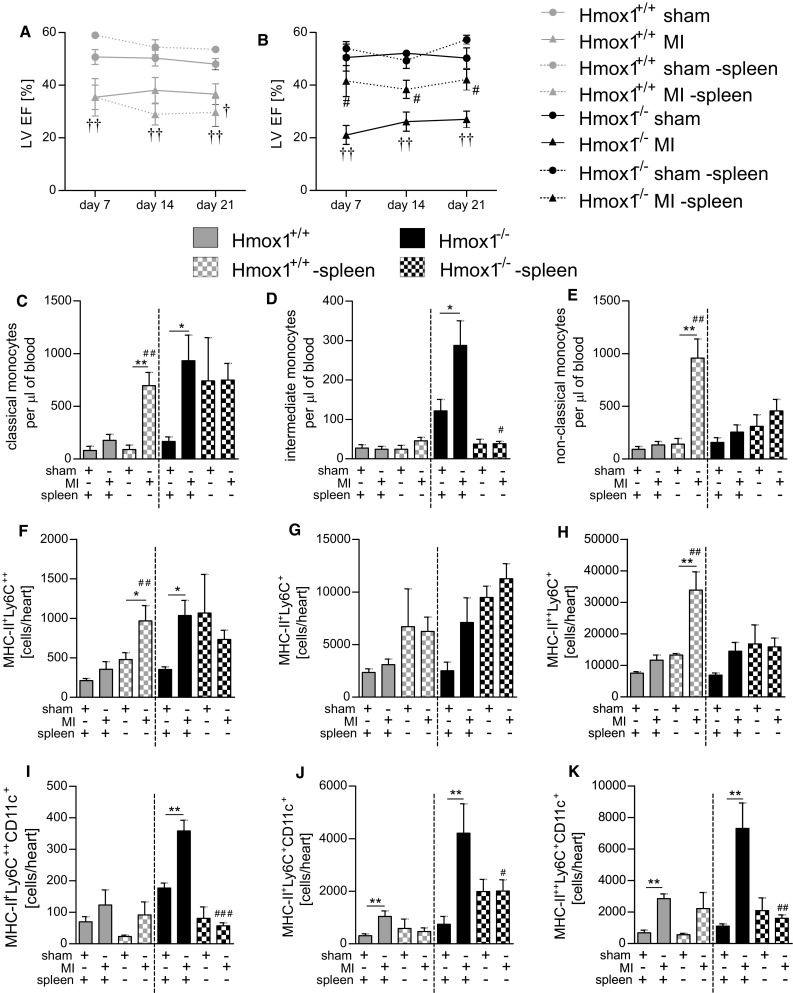
Splenectomy changes the post-MI patterns of monocytes/macrophages and LV function in Hmox1^+/+^ and Hmox1^−/−^ mice. LV EF in splenectomized vs. non-splenectomized **a** Hmox1^+/+^ and **b** Hmox1^−/−^ mice monitored for 21 days post-sham or -MI surgery. Flow cytometric analysis of **c** classical (CD45^+^ CD11b^+^ Ly6G^−^ NK1.1^−^ Ly6C^++^ CD43^+^), **d** intermediate (CD45^+^ CD11b^+^ Ly6G^−^ NK1.1^−^ Ly6C^++^ CD43^++^) and **e** non-classical (CD45^+^ CD11b^+^ Ly6G^−^ NK1.1^−^ Ly6C^+^ CD43^++^) monocytes in the blood. Subsets of cardiac macrophages: **f** MHC-II^+^ Ly6C^++^, **g** MHC-II^+^ Ly6C^+^, **h** MHC-II^++^ Ly6C^+^, **i** MHC-II^+^ Ly6C^++^ CD11c^+^, **j** MHC-II^+^ Ly6C^+^ CD11c^+^, **k** MHC-II^++^ Ly6C^+^ CD11c^+^ at 21 days after LAD ligation or sham surgery (*n* = 3–9 mice/group). Data represented as **c**–**e** a number of cells per 1 µl of peripheral blood and **f**–**k** a number of cells detected in heart. *p < 0.05, **p < 0.01, ***p < 0.0001. ^#^vs. corresponding cardiac surgery in non-splenectomized mice

We next evaluated animals splenectomized one month before either sham or MI surgeries. Removal of spleen differently affected the LV EF in Hmox1^+/+^ and Hmox1^−/−^ mice starting from 7th until 21st day post-MI (Fig. 9a, b). In splenectomized WT mice there was a trend towards greater reduction of post-MI LV function than in non-splenectomized individuals of the same genotype subjected to LAD ligation (Fig. 9a). Opposite effect was observed in Hmox1-deficient mice where splenectomy was associated with significantly improved post-MI LV EF (Fig. 9b) suggesting an important role of spleen in post-MI heart failure progression in Hmox1^−/−^ mice.

Next, we analyzed different subsets of blood monocytes and cardiac macrophages in splenectomized animals 21 days after LAD ligation. In Hmox1^+/+^ mice removal of spleen resulted in significantly increased post-MI numbers of classical (Fig. 9c) and non-classical (Fig. 9e) monocytes, while intermediate monocytes (Fig. 9d) remained unchanged when compared to sham-operated splenectomized controls. Flow cytometric analysis of myocardium revealed significantly increased MHC-II^+^ Ly6C^++^ (Fig. 9f) and MHC-II^++^ Ly6C^+^ (Fig. 9h) macrophage subsets in splenectomized WT mice after MI. Among all analyzed macrophage populations the CD11c^+^ subsets remained unchanged in MI-operated Hmox1^+/+^ mice when compared to non-splenectomized individuals of the same genotype subjected to LAD ligation.

Removal of spleen in Hmox1^−/−^ mice did not significantly change the post-MI numbers of neither classical (Fig. 9c) nor non-classical (Fig. 9e) blood monocytes when compared to sham-operated splenectomized controls 21 days post-surgery. Interestingly, however, the numbers of intermediate monocytes (Fig. 9d) were markedly reduced after spleen removal (both in sham- and MI-operated Hmox1^−/−^ individuals). Flow cytometric analysis of different subpopulations of cardiac macrophages revealed that there were no changes in the numbers of MHC-II^+^ Ly6C^++^ (Fig. 9f), MHC-II^+^ Ly6C^+^ (Fig. 9g) and MHC-II^++^ Ly6C^+^ (Fig. 9h) subsets in splenectomized Hmox1^−/−^ mice with MI when compared to non-splenectomized individuals of the same genotype subjected to LAD ligation. Concomitantly, we observed a potent reduction of MHC-II^+^ Ly6C^++^ CD11c^+^ (Fig. 9i), MHC-II^+^ Ly6C^+^ CD11c^+^ (Fig. 9j) and MHC-II^++^ Ly6C^+^ CD11c^+^ (Fig. 9k) macrophage subsets following removal of spleen in MI-operated Hmox1^−/−^ mice when compared to non-splenectomized individuals of the same genotype subjected to MI.

## Discussion

Following cardiac ischemia, the immune system has an important and complex role in driving the healing of injured myocardium. Better understanding of these multi-faceted cellular and molecular interactions may help to prevent the unwanted inflammatory damage, adverse cardiac remodeling and heart failure after ischemic episode.

Here we report that Hmox1 has a dual role in MI. On one hand, its deficiency is associated with better overall survival in the first 3–5 days post-ischemia due to much lower incidence of LVFWR. On the other hand, within the next few days Hmox1^−/−^ animals develop greater LV dysfunction than the surviving Hmox1^+/+^ mice. This phenomenon was associated with increased post-MI mobilization of inflammatory Ly6C^hi^ monocytes. Their increased cardiac recruitment resulted in a greater abundance of inflammatory MHC-II^++^ Ly6C^+^ CD11c^+^ macrophages in the hearts of Hmox1-deficient mice. Importantly, removal of splenic reservoir of monocytes limited the adverse late post-ischemic LV remodeling in Hmox1^−/−^ mice.

In this study we used two methods to confirm the efficacy of the MI surgery and comparable in both genotypes initial infarct size. Measurement of hypoxic cells with hypoxia marker (pimonidazole hydrochloride) enables post-mortem analysis as early as 90 min after ischemia [24, 26] and may constitute a good alternative for histological methods determining the infarct size, i.e., triphenyltetrazolium chloride (TTC) staining [2]. The use of hypoxyprobe showed comparable numbers of hypoxic cells in the hearts of WT and Hmox1-deficient mice shortly after LAD ligation. The other measurement, evaluation of cTnI in the peripheral blood, was even more useful as mice with improperly induced MI, what sometimes happens due to anatomical variability of the left coronary artery branches [1], had cTnI at the level of sham-operated animals and thus were excluded from further investigation.

Previous studies demonstrated the cardioprotective action of Hmox1 in both short-term [22, 54, 55] (I/R) and long-term [47] (permanent LAD ligation) ischemic cardiac stress. This can be attributed to the antioxidant, anti-apoptotic, and anti-inflammatory effects of the end-products of heme catabolism (biliverdin/bilirubin and CO) [7, 34]. Additionally, Hmox1 may also act independently of enzymatic function, as its truncated form is found in the nucleus where it can regulate transcription factors involved in the antioxidant response [31]. In our study, *Hmox1* gene was strongly upregulated in the hearts of WT mice 1 day after MI. Then, the expression decreased with time reaching the baseline on day 21 post-surgery. In fact, *Hmox1* gene contains one of the most regulated promoters identified so far. In addition to its substrate heme, several other compounds can also upregulate *Hmox1* expression, just to mention reactive oxygen species, inflammatory cytokines or hypoxia in rodents [33]. All of these above-mentioned appear and can induce *Hmox1* expression under ischemic stress.

In the present study, we observed much lower occurrence of LVFWR and better overall survival in the first 3–5 days after permanent LAD ligation in mice deprived of Hmox1. According to clinical data, cardiac rupture most often occurs in patients with a large ST elevation MI (STEMI) and over the past few decades its occurrence has been greatly reduced due to higher use of reperfusion procedures and pharmacotherapy [17]. Thus, the observed difference is mostly related to our experimental model of MI associated with a prolonged ischemia, whereas in short-term cardiac stress following I/R injury such differences may be covert. In animals, the likelihood of rupture after MI depends on species, strain, gender, and age (reviewed in Ref. [42]). Experimental studies identified several gene products influencing rates of cardiac rupture (reviewed in Ref. [42]). Among these, decreased collagen content has been associated with infarct expansion and rupture [5, 18, 19, 50]. Hmox1 was previously described as vulnerable atherosclerotic plaque defining agent [6] and its overexpression with gene therapy decreased interstitial collagen in rat hearts exposed to I/R injury [32]. Moreover, it was recently shown that Hmox1^−/−^ mice exhibit a higher than WT mice degree of collagen deposition in their atria [52]. To the best of our knowledge, here we report for the first time that the lack of Hmox1 significantly lowers the risk of LVFWR after permanent LAD ligation. The protective effect on this early rapid and deadly LV remodeling was associated with greater than in WT mice collagen type I production in the damaged area of the heart.

Next, starting from 7th until 21st day post-MI with TTE we observed an opposite effect—significantly impaired LV function in Hmox1^−/−^ mice, greater than in WT individuals. WGA staining performed 21 days after MI revealed greater cardiomyocyte hypertrophy in the LV peri-infarct zone of Hmox1-deficient mice than in their WT littermates. Hmox1 was previously shown to modulate the late post-MI remodeling by attenuation of LV hypertrophy and interstitial fibrosis [38, 47]. These beneficial effects of Hmox1, and particularly one of its reaction product—CO, are at least partially mediated by regulation of cell cycle of different cells residing the heart—vascular smooth muscle cells (VSMCs), fibroblasts, endothelial cells and cardiomyocytes [28, 30, 32, 48]. Additionally, cardiac monocyte-derived macrophages may intensify the post-MI LV remodeling and heart failure due to excessive immune response [29].

Increased number of classical Ly6C^hi^ monocytes circulating in the blood of Hmox1-deficient mice and their enhanced post-I/R cardiac influx was recently associated with increased infarct size and impaired LV wall motion [22]. In the present study we also detected greater numbers of inflammatory classical and intermediate Ly6C^hi^ monocytes in the blood of Hmox1^−/−^ when compared to Hmox1^+/+^ mice both at steady state and 21 days post-MI. In addition, this was associated with prolonged upregulation of cardiac *Mcp*-*1* gene expression, greater induction of adhesion molecules and increased numbers of monocyte-derived macrophages in the hearts of Hmox1-deficient mice with MI. Particularly, we noticed expansion of MHC-II^+^ Ly6C^++^ macrophage population starting from 4th until 21st day post-MI. It is well established that Ly6C^hi^ cells are the predominant subtype during the first few days post-MI, after which time Ly6C^lo^ macrophages begin to predominate [37]. Additionally, among MHC-II^+^ Ly6C^++^, MHC-II^+^ Ly6C^+^ and MHC-II^++^ Ly6C^+^ macrophages, the CD11c^+^ subsets expanded. On day 21, there was a significant difference in the number of all analyzed CD11c^+^ macrophage populations between WT and Hmox-1-deficient mice subjected to MI. Contrary to cardiac-resident macrophages, the monocyte-derived macrophages were shown to strongly express on their surface CD11c antigen [14]. The CD11c^+^ macrophages represent a classical proinflammatory phenotype capable of efficiently producing IL-1β, TNF-α and IFN-γ [23]. Moreover, elevated levels of MHC II (and other co-stimulatory molecules) on the surface (MHC^++^) make these cells highly professional antigen-presenting cells capable of promoting strong lymphocyte responses [23]. Expansion of these proinflammatory macrophage subsets in the infarcted hearts of Hmox1-deficient mice indicates their involvement in the heart failure progression.

Hmox1 is a critical regulator of the balance between self-renewal and differentiation of HSPCs under environmental stress as its haplodeficiency (Hmox1^+/−^) was associated with premature depletion of HSC reserve following repeated myelotoxic injuries [4]. In the bone marrow of Hmox1-deficient mice at steady state we detected more numerous populations of all investigated HSPCs. Moreover, GMPs on day 4 and SKL, ST-HSC, MPP on day 21 post-MI were more abundant in the bone marrow of Hmox1^−/−^ than Hmox1^+/+^ mice. This indicates a more active hematopoiesis in the bone marrow of Hmox1-deficient mice subjected to MI.

In addition to bone marrow [12], spleen may contribute to the post-MI monocyte mobilization. This organ was described as a site for storage and rapid deployment of monocytes [45]. In this way, injury-induced monocytopoiesis usually does not lead to serious disturbances in generation of lymphocytes, erythrocytes and platelets in the bone marrow. In the spleen of intact Hmox1-deprived animals we detected higher numbers of different monocyte subsets—especially classical and intermediate which were significantly increased. On day 21 post-MI the numbers of classical and intermediate monocytes in spleens of Hmox1^−/−^ mice were lower than in their WT littermates. At the same time after MI, the numbers of these two monocyte subsets were increased in the blood of Hmox1-deficient mice, what may be a consequence of their departure from the spleen.

It was previously shown that following MI, upstream progenitors are mobilized from the bone marrow niches and engrafted in the spleen [11, 40]. Activation of such supportive hematopoiesis leads to generation of surplus circulating monocytes. Analysis of different populations of HSPCs in spleen revealed an increase of SKLs and, among them LT-HSCs, on 1st day following MI in spleens of mice of both genotypes. Then, their numbers markedly dropped on day 4 indicating their exploitation in extramedullary hematopoiesis in the spleen. What is more, significantly lower steady state numbers of GMPs in Hmox1^−/−^ than Hmox1^+/+^ mice were found. All these observations support a hypothesis, that the process of generation and liberation of monocytes from the spleens of Hmox1^−/−^ mice after MI is so intense, that in a consequence it leads to a rapid exhaustion of splenic reserve of hematopoietic progenitors.

We finally demonstrated an important role of spleen in the post-MI mobilization of the proinflammatory Ly6C^hi^ monocytes and heart failure progression in splenectomized Hmox1^−/−^ mice. Splenectomy significantly decreased the numbers of intermediate Ly6C^hi^ monocytes and prevented the post-MI expansion of the two inflammatory Ly6C^hi^ (classical and intermediate) subsets in the blood of Hmox1-deficient mice. As a consequence, the post-MI numbers of proinflammatory monocyte-derived macrophages, including CD11c^+^ subsets of all analyzed macrophage populations were decreased in the hearts of splenectomized Hmox1^−/−^ mice and their heart function improved significantly when compared to non-splenectomized MI-operated Hmox1-deficient individuals. In contrast, removal of spleen in Hmox1^+/+^ mice resulted in excessive post-MI numbers of classical Ly6C^hi^ and non-classical Ly6C^lo^ monocytes, increased numbers of monocyte-derived cardiac macrophages (but not in the CD11c^+^ subsets) and a tendency towards greater LV dysfunction when compared to non-splenectomized MI-operated WT mice. Such discrepancies between splenectomized Hmox1^+/+^ and Hmox1^−/−^ mice may suggest that Ly6C^hi^ classical and intermediate monocytes may have different origin. Intermediate monocytes in Hmox-1-deficient mice seem to derive from the spleen, as their numbers are dramatically reduced following splenectomy. A potent increase in the numbers of classical monocytes in the blood of splenectomized WT mice after MI indicates their bone marrow origin. This can further implicate different function of these cells in the injured cardiac muscle. In fact, Dutta et al. described different mRNA profile in monocytes of splenic and bone marrow origin and shown similarities in inflammatory gene expression between monocytes isolated from the spleen and Ly6C^hi^ monocytes isolated from atherosclerotic plaque after MI [11].

A limitation of this study is that the mechanism by which Hmox1 regulates the production of monocytes still remains to be elucidated. The monocyte-macrophage lineage commitment at steady-state and during inflammation undergoes a tight control intrinsically via several transcription factors and extrinsically via different cytokines/growth factors [44]. Thus, it would be interesting to investigate some of these mechanisms in the future.

In conclusion, our findings demonstrate a dual role of Hmox1 in MI. On one hand Hmox1 deficiency improves early post-MI survival by decreasing the occurrence of LVFWR. Afterwards, however, the absence of Hmox1 is associated with adverse late LV remodeling and severe heart failure after MI. This was associated with an increased monocyte mobilization to the blood and exaggerated myocardial macrophage infiltration. We identified spleen as an important source of the surplus circulating inflammatory Ly6C^hi^ monocytes contributing to the severe LV dysfunction after MI in Hmox1^−/−^ mice. Our findings may indicate that any therapeutic strategies aimed at modulation of Hmox1 expression in the heart post-MI should be applied in a timely manner.

## Electronic supplementary material

Below is the link to the electronic supplementary material.

Supplementary material 1 (DOC 55 kb)

Supplementary material 2 (PDF 166 kb)

Supplementary material 3 (PDF 472 kb)

Supplementary material 4 (PDF 1128 kb)
