# Supplementary material for: Splenic Ly6Chi monocytes contribute to adverse late post-ischemic left ventricular remodeling in heme oxygenase-1 deficient mice
Source: Basic Res Cardiol. 2017 May 22;112(4):39. doi: 10.1007/s00395-017-0629-y (PMC5440541; doi:10.1007/s00395-017-0629-y)
Supplement: Supplementary file 1 — Supplementary material 1 (DOC 55 kb) [file 395_2017_629_MOESM1_ESM.doc]

Supplemental Material for

**Splenic Ly6C^hi^ monocytes contribute to adverse late post-ischemic left ventricular remodeling in heme oxygenase-1 deficient mice**

Mateusz Tomczyk^1^, Izabela Kraszewska^1^, Krzysztof Szade^1^, Karolina Bukowska-Strakova^1,2^, Marco Meloni^3^, Alicja Jozkowicz^1^, Jozef Dulak^1,4^, Agnieszka Jazwa^1^

^1^Department of Medical Biotechnology, Faculty of Biochemistry, Biophysics and Biotechnology, Jagiellonian University, Krakow, Poland; ^2^Department of Clinical Immunology and Transplantology, Polish-American Institute of Pediatrics, Medical College, Jagiellonian University Krakow, Poland; ^3^British Heart Foundation Centre for Cardiovascular Science, University of Edinburgh, UK; ^4^Malopolska Centre of Biotechnology, Jagiellonian University, Krakow, Poland

**Supplemental Table 1.** Antibodies used for detection of monocytes and macrophages with flow cytometry.

| **Antigen** | **Format** | **Clone** |
| --- | --- | --- |
| NK1.1 | BV605 | PK136 |
| Ly-6G | BV605 | 1A8 |
| CD45 | BV786 | 30-F11 |
| CD11b | PE-CF549 | M1/70 |
| CD11c | AlexaFluor700 | N418 |
| Ly-6C | PerCP-Cyanine5.5 | HK1.4 |
| CD43 | PE | S7 |
| MHC II | PE-Cyanine7 | M5/114.15.2 |

**Supplemental Table 2.** Antibodies used for detection of hematopoietic stem and progenitor cells with flow cytometry.

| **Antigen** | **Format** | **Clone** |
| --- | --- | --- |
| CD3 | PE | 17A2 |
| Gr-1 |  | RB6-8C5 |
| B220 |  | RA3-6B2 |
| Ter-119 |  | TER-119 |
| CD11b |  | M1/70 |
| CD117 (c-kit) | APC-eFluor 780 | 2B8 |
| Sca-1 | PE-Cy7 | D7 |
| CD34 | Alexa Fluor 700 | RAM34 |
| CD48 | PerCP-Cyanine5.5 | HM48-1 |
| CD150 | APC | TC15-12F12.2 |

**Supplemental Table 3.** Primers used for detection of mRNA transcripts.

| **Transcript** | **Primer sequence 5’→3’** | **Annealing temperature [^o^C]** |
| --- | --- | --- |
| Heme oxygenase-1 (*Hmox1*) | Forward: CCTCACTGGCAGGAAATCATC  Reverse: CCTCGTGGAGACGCTTTACATA | 59 |
| Monocyte chemoattractant protein-1 (*Mcp1*) | Forward: CCCAATGAGTAGGCTGGAGA  Reverse: TCTGGACCCATTCCTTCTTG | 58 |
| E-Selectin (*E*s*el*) | Forward: ATGCCTCGCGCTTTCTCTC  Reverse: GTAGTCCCGCTGACAGTATGC | 58 |
| Vascular cell adhesion molecule-1 (*Vcam1*) | Forward: CCGGCATATACGAGTGTGAA  Reverse: GATGCGCAGTAGAGTGCAAG | 56 |
| Intracellular adhesion molecule-1 (*Icam1*) | Forward: GAGCTCGAGAGTGGACCCAA  Reverse: CAGGGTGAGGTCCTTGCCTAC | 61 |
| Elongation factor 2 (*Ef2*) | Forward: TCAGCACACTGGCATAGAGGC  Reverse: GACATCACCAAGGGTGTGCAG | 60 |

**Supplemental figure legends**

**Supplemental Figure 1.** Gating strategy for monocytes in peripheral blood and spleen.

**Supplemental Figure 2.** Gating strategy for cardiac monocytes and macrophages.

**Supplemental Figure 3.** Gating strategy for hematopoietic stem and progenitor cells in bone marrow and spleen.
