# Supplementary figures and images for: Splenic Ly6Chi monocytes contribute to adverse late post-ischemic left ventricular remodeling in heme oxygenase-1 deficient mice
Source: Basic Res Cardiol. 2017 May 22;112(4):39. doi: 10.1007/s00395-017-0629-y (PMC5440541; doi:10.1007/s00395-017-0629-y)

# Supplemental Figure 1

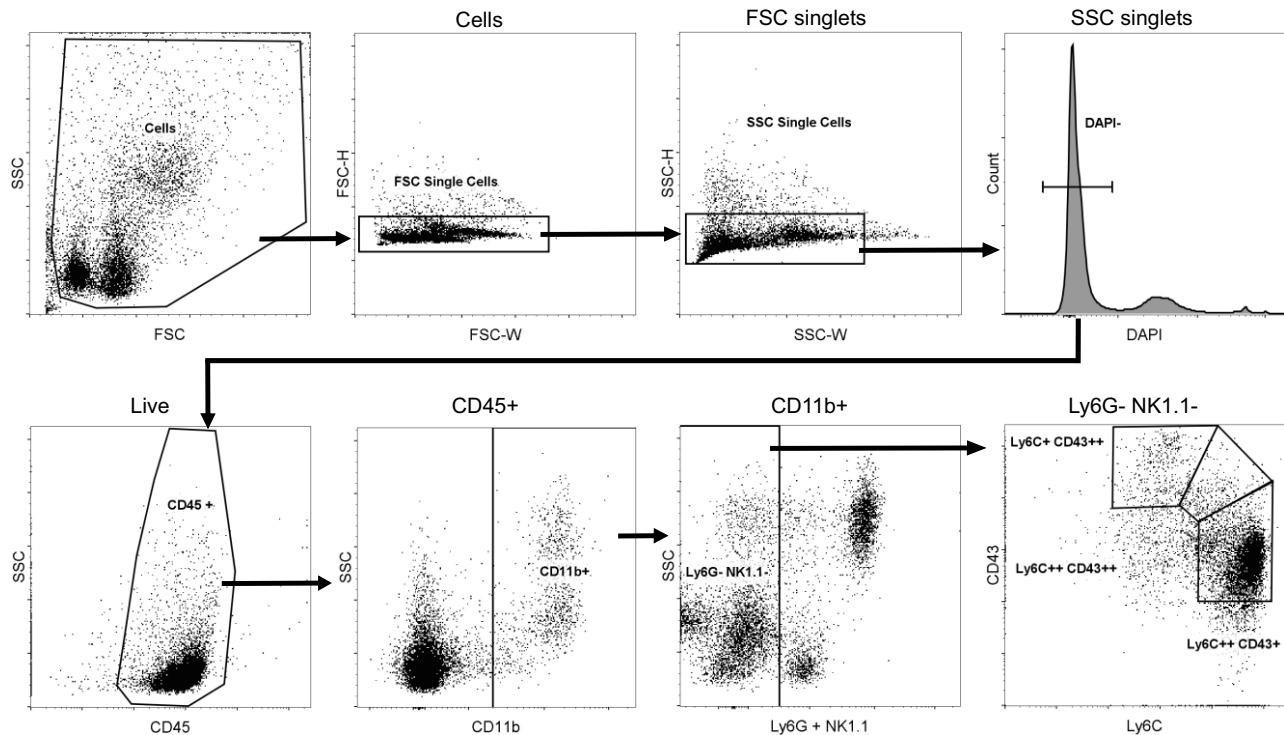

Supplement: Supplementary file 2 — Supplementary material 2 (PDF 166 kb) [file 395_2017_629_MOESM2_ESM.pdf]

## Supplemental Figure 2

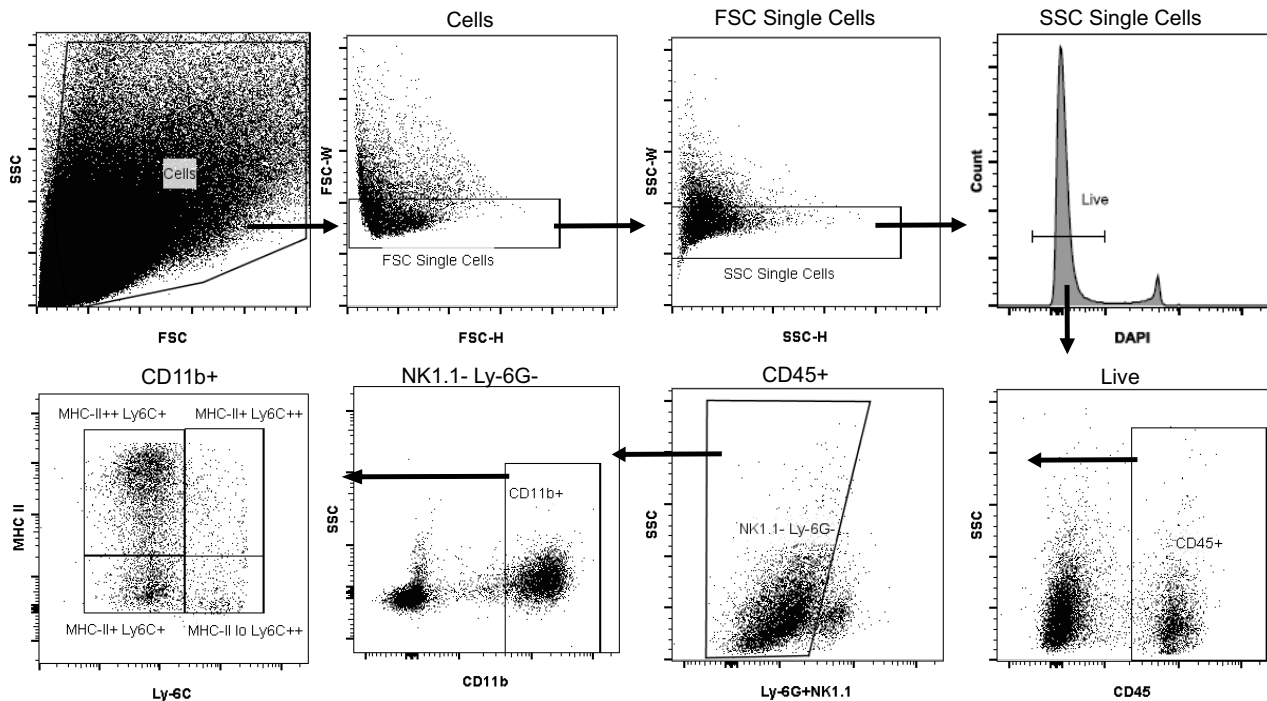

Supplement: Supplementary file 3 — Supplementary material 3 (PDF 472 kb) [file 395_2017_629_MOESM3_ESM.pdf]

# Supplemental Figure 3

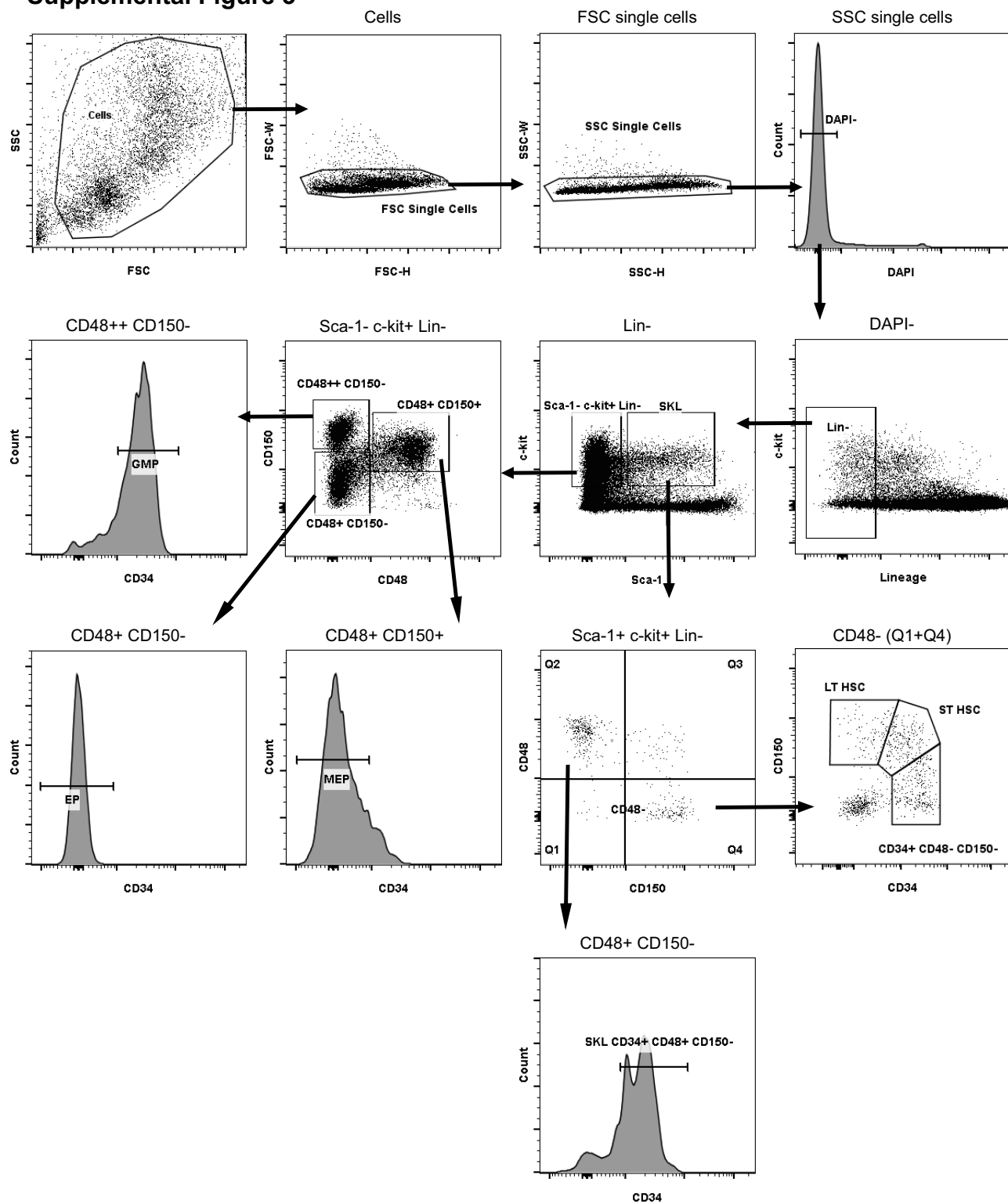

Supplement: Supplementary file 4 — Supplementary material 4 (PDF 1128 kb) [file 395_2017_629_MOESM4_ESM.pdf]
